# Becker muscular dystrophy caused by exon 2-truncating mutation of *DMD*

**DOI:** 10.1038/s41439-019-0083-5

**Published:** 2019-11-18

**Authors:** Tetsuhiko Ikeda, Hidehiko Fujinaka, Kiyoe Goto, Takashi Nakajima, Tetsuo Ozawa

**Affiliations:** 1 Department of Neurology, National Hospital Organization Niigata National Hospital, Kashiwazaki, Niigata Japan; 2 Department of Neurology, Brain Research Institute, Niigata University, Niigata, Niigata Japan; 3 Department of Pediatrics, National Hospital Organization Niigata National Hospital, Kashiwazaki, Niigata Japan; 4 Department of Clinical Research, National Hospital Organization Niigata National Hospital, Kashiwazaki, Niigata Japan; 5 Deprtment of Genetic Counseling, National Hospital Organization Niigata National Hospital, Kashiwazaki, Niigata Japan; 6 Department of Neurology, National Hospital Organization Niigata National Hospital, Kashiwazaki, Niigata Japan; 7 Department of Internal Medicine, National Hospital Organization Niigata National Hospital, Kashiwazaki, Niigata Japan

**Keywords:** Cancer epigenetics, Microbial genetics

## Abstract

Nonsense and frameshift mutations of the dystrophin (*DMD*) gene usually cause severe Duchenne muscular dystrophy (DMD). Interestingly, however, premature stop codons in exons 1 and 2 result in relatively mild Becker muscular dystrophy (BMD). Herein, we report the clinical course of a patient with a very mild phenotype of BMD caused by a frameshift mutation, NM_004006.2: c.40_41del GA/p.(Glu14ArgfsX17), in exon 2 of the *DMD* gene.

The dystrophin (*DMD*) gene, located at Xp21.2-p21.1, is one of the largest human genes and consists of 79 exons. The *DMD* gene encodes dystrophin, a large rod-shaped protein that lies on the inner side of the skeletal and cardiac muscle cell membrane. Dystrophin assembles with various proteins to form the dystrophin-associated protein complex (DAPC). The DAPC plays a critical role in stabilizing the plasma membrane of striated muscle by linking the actin cytoskeleton to the extracellular matrix.

Loss-of-function mutations in the *DMD* gene result in the following two common forms of X-linked recessive muscular dystrophy: the more severe form of Duchenne muscular dystrophy (DMD) and the milder form of Becker muscular dystrophy (BMD). In DMD, progressive muscular weakness usually develops in early childhood between 2 and 3 years of age, progresses to the wheelchair-bound stage by the age of 12 years, and might result in death at 30 years of age because of respiratory insufficiency or heart failure. Patients with BMD show similar signs and symptoms of DMD with a later onset and very broad spectrum of phenotypes, ranging from a “near DMD” to an almost asymptomatic state. These phenotypes generally depend on the amount of functional dystrophin protein in muscle cells. In DMD, the dystrophin protein is completely absent or present in very small amounts (<3% of normal levels)^1^ due to frameshift mutations, whereas in BMD, the dystrophin protein is partially functional (reduced amount or truncated size) due to in-frame (preserved reading frame) large-scale mutations or missense mutations. However, exceptions to this reading-frame rule have been observed in ~10% of patients with DMD and BMD^2–5^. Deviation from the reading-frame rule is thought to involve multiple molecular mechanisms, which are not fully explained. Herein, we report the clinical course of a patient with a two-base deletion mutation of the *DMD* gene, which causes a frameshift and results in a premature stop codon in exon 2. This truncating mutation is expected to terminate the translation of the gene in the N-terminal actin-binding domain and cause the complete absence of muscular dystrophin. However, interestingly, this patient showed a very mild phenotype of BMD.

A 61-year-old Japanese man with muscular dystrophy visited the Clinical Genetics Outpatient Department of Niigata National Hospital seeking a more precise diagnosis by genetic testing. His muscular symptoms first manifested as toe running and pseudohypertrophy of the calves around the age of 10 years. He became aware of his difficulty in climbing stairs beginning at the age of 20 years. At the age of 28 years, he was admitted to a university hospital and diagnosed with sporadic BMD based on his clinical symptoms, serum creatine kinase elevation, and dystrophic changes in his muscle biopsy specimens. However, immunostaining for the muscular dystrophin protein was not available because the *DMD* gene had not been discovered at that time. A few decades after the first diagnosis of BMD, his attending doctor pointed out the possibility that his illness might not be BMD, but limb-girdle muscular dystrophy (LGMD) because the progression of his clinical symptoms was relatively slower than expected. He was intellectually normal. He could walk until the age of 53 years and move independently with his buttocks on the floor up to the age of 60 years. He showed no symptoms of heart failure at the age of 61 years. To resolve this ambiguity, he desired to obtain a definitive diagnosis by genetic testing. During the pretest genetic counseling, we obtained a detailed family history, which revealed that three of his maternal male cousins had mild muscle weakness, although they were not diagnosed with muscular disease. This information strongly suggested that his disease followed an X-linked recessive pattern of inheritance. After obtaining written informed consent, we performed genetic testing for both BMD and LGMD. We analyzed the *DMD* gene using multiplex ligation-dependent probe amplification analysis (MLPA, MRC-Holland). In addition, we analyzed the *DMD* gene and the major genes responsible for LGMD by next-generation sequencing (NGS) using a selected exome panel (TruSight One; Illumina). The MLPA results revealed no exonic deletions/duplications of the *DMD* gene, whereas NGS revealed a two-base deletion in exon 2 of the *DMD* gene, which caused a frameshift and premature stop codon (NM_004006.2: c.40_41del GA/p.(Glu14ArgfsX17)). This mutation was confirmed by Sanger sequencing (Fig. 1). We analyzed the 34 primary genes causing LGMD or LGMD-like diseases using NGS. However, no pathogenic variant was detected.

**Fig. 1:**
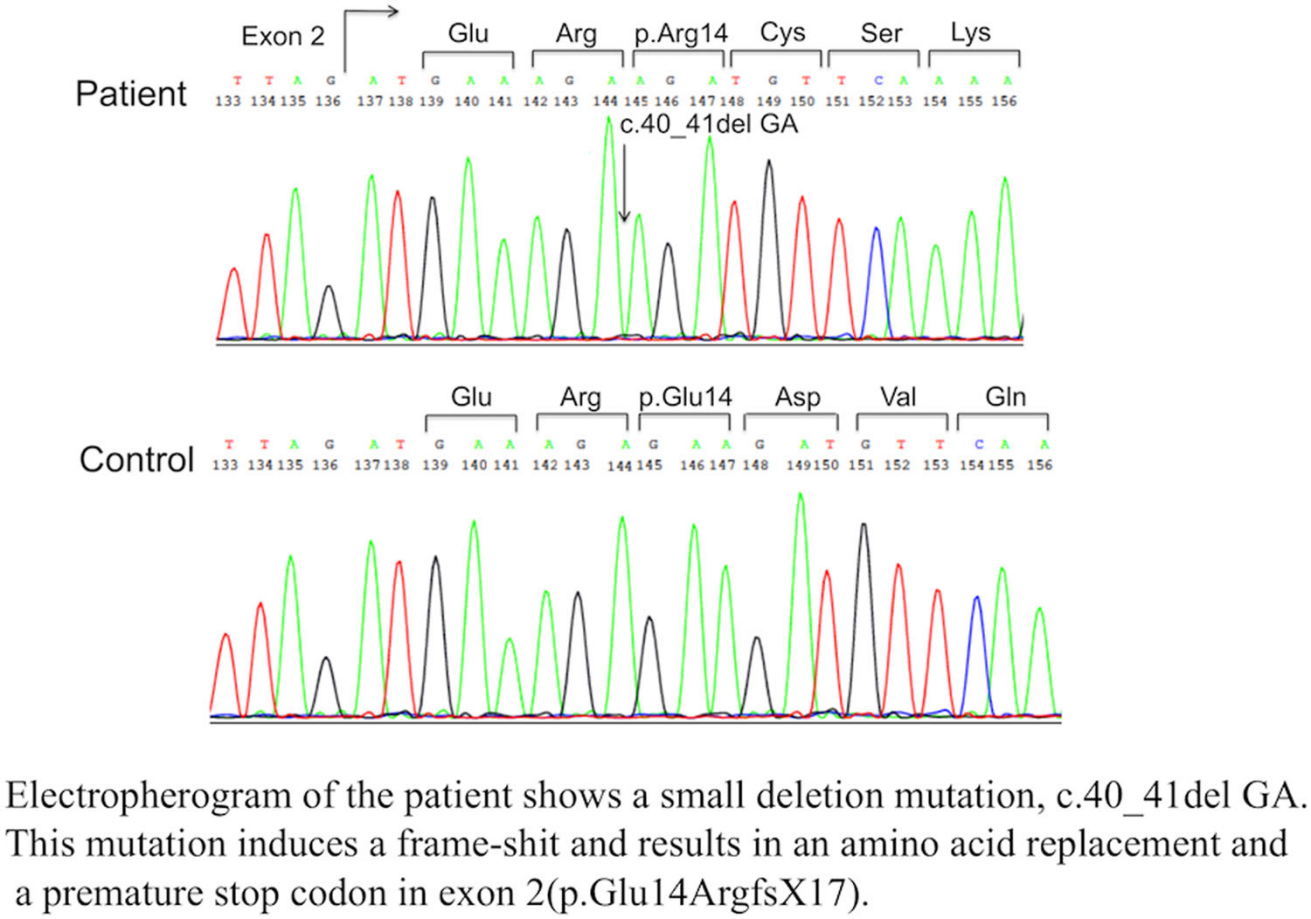
Sanger sequencing of the mutation site. Electropherogram of the patient shows a small deletion mutation, c.40_41del GA. This mutation induces a frame shit and results in an amino acid replacement and a premature stop codon in exon 2(p.Glu14ArgfsX17)

Limb-girdle muscular dystrophy is a descriptive term for a group of muscular dystrophies, exclusive of DMD/BMD, that manifests as weakness and muscle wasting predominantly in the proximal portion of the arms and legs. The genetic background of LGMD is highly heterogeneous. To date, more than 50 responsible genes and loci have been reported. Type 1 LGMD is inherited in an autosomal dominant manner, and type 2 LGMD (LGMD2) is caused by autosomal recessive mutations. Because several genes related to LGMD2 encode the key components of the DAPC, such as α-, β-, γ-, and δ-sarcoglycan, patients with LGMD2 usually have phenotypes similar to those of BMD. In this study, we investigated the LGMD-related genes included in TruSight One by NGS, but found no genetic changes causing the illness in the patient.

In contrast, we found that this patient had a truncating mutation p.(Glu14ArgfsX17) in exon 2 of the *DMD* gene. This mutation is very rare but is already listed in the mutational databases such as the Human Genome Mutation Database (http://www.hgmd.cf.ac.uk/ac/index.php) and the Leiden Muscular Dystrophy pages (https://www.dmd.nl/) as a mutation causing the BMD phenotype. The diagnosis of BMD was confirmed based on the results of genetic testing.

Multiple molecular mechanisms are thought to be involved in the exceptions to “the reading-frame rule” in DMD/BMD. For example, severe phenotypes of patients with DMD with in-frame exonic deletion mutations can be explained by a very large deletion that produces a dysfunctional dystrophin or the loss of a functionally essential portion of dystrophin, such as the actin-binding site^4^. Milder phenotypes of patients with mutations in the 5′ region of the *DMD* gene have also been reported as exceptions to the rule^3,6^. In these cases, alternative splicing is considered a factor leading to these exceptions^3,7^.

Recently, another notable mechanism of phenotypic amelioration was proposed.

It is known that premature stop codons in exon 1 of the *DMD* gene, such as p.(Trp3X) and p.(Glu5ValfsX3), resulted in very mild phenotypes of BMD. The first reported case of p.(Trp3X) showed no symptoms until age 20 years and could walk until 62 years of age^8^. The patient with p.(Glu5ValfsX3) was ambulant until 42 years of age^6^. Gurvich et al.^6^ revealed that these truncating mutations in exon 1 induced alternative translation initiation at two AUG codons within exon 6 and resulted in considerable amelioration of the phenotype. Furthermore, Wein et al.^9^ demonstrated that the reinitiation of translation of exon 6 was mediated by the activation of an internal ribosomal entry site within exon 5 of the *DMD* gene. Internal ribosomal entry sites are translation regulatory sequences that govern cap-independent translation initiation, which is activated when cap-dependent translation is compromised in eukaryotes. This altered translation initiation mechanism is thought to be applied to the premature stop codons in exons 1 and 2 of the *DMD* gene, such as p.(Trp3X), p.(Glu5ValfsX3), and p.(Glu14ArgfsX17), which is the same mutation found in our patient. They also demonstrated that the N-terminus truncated isoforms of dystrophin derived from these premature stop codons were functional and that they were expressed in the muscles of individuals with such mutations at decreased levels. These findings indicate the potential for a new therapeutic approach that promotes the initiation of translation in exon 6 for patients with mutations in the early *DMD* exons^9^.

The clinical symptoms of our patient are consistent with the theoretical explanation presented by Wein et al.^9^. This is not the first report of a p.(Glu14ArgfsX17) mutation in the *DMD* gene. However, we believe that our findings are worth reporting because the detailed symptoms and clinical course of an individual with this mutation have not been described^6,9^.

## Data Availability

The relevant data from this Data Report are hosted at the Human Genome Variation Database at 10.6084/m9.figshare.hgv.2792.
